# Image-Based Structural Modeling of the Cardiac Purkinje Network

**DOI:** 10.1155/2015/621034

**Published:** 2015-10-25

**Authors:** Benjamin R. Liu, Elizabeth M. Cherry

**Affiliations:** School of Mathematical Sciences, Rochester Institute of Technology, Rochester, NY 14623, USA

## Abstract

The Purkinje network is a specialized conduction system within the heart that ensures the proper activation of the ventricles to produce effective contraction. Its role during ventricular arrhythmias is less clear, but some experimental studies have suggested that the Purkinje network may significantly affect the genesis and maintenance of ventricular arrhythmias. Despite its importance, few structural models of the Purkinje network have been developed, primarily because current physical limitations prevent examination of the intact Purkinje network. In previous modeling efforts Purkinje-like structures have been developed through either automated or hand-drawn procedures, but these networks have been created according to general principles rather than based on real networks. To allow for greater realism in Purkinje structural models, we present a method for creating three-dimensional Purkinje networks based directly on imaging data. Our approach uses Purkinje network structures extracted from photographs of dissected ventricles and projects these flat networks onto realistic endocardial surfaces. Using this method, we create models for the combined ventricle-Purkinje system that can fully activate the ventricles through a stimulus delivered to the Purkinje network and can produce simulated activation sequences that match experimental observations. The combined models have the potential to help elucidate Purkinje network contributions during ventricular arrhythmias.

## 1. Introduction

Cardiac arrhythmias are disruptions in the normal electrical activity of the heart. The heart functions through mechanical contraction, which is a byproduct of electrical excitation of cardiac tissue. Disruptions in this electrical activity can therefore compromise the contraction of the cardiac muscle, leading to ineffective pumping of blood. While some types of arrhythmias have little noticeable effect, others are potentially lethal. Ventricular arrhythmias are especially dangerous because of the critical role the ventricles play in normal heart rhythm. The ventricles are thick, muscular chambers that actively pump blood out of the heart and throughout the body. Ventricular arrhythmias can rapidly become life-threatening if normal electrical activity is not restored, which usually requires the use of aggressive techniques such as defibrillation.

Within the ventricles, a specialized conduction system called the Purkinje network serves to facilitate the timely and correct sequence of activation of the ventricles. The Purkinje network functions to distribute a stimulus throughout the entire ventricles, leading to a much faster total activation of the ventricles than could be achieved by the spread of excitation solely through the ventricular muscle. The role of the Purkinje network is crucial during normal heart rhythm, but much less is known regarding its function during ventricular arrhythmias. It is known that electrophysiological characteristics of Purkinje cells, such as enhanced normal automaticity, abnormal automaticity, triggered activity, and electrical alternans, may be proarrhythmic [1–4]. Here, we are motivated by experiments that have shown that the Purkinje network itself may be important to the genesis and maintenance of certain types of arrhythmias [5–7] and that the absence of the network can alter arrhythmia dynamics [8], but further study is needed to improve our understanding of the effect of the Purkinje network during such events.

A number of previous modeling efforts have been made to incorporate Purkinje-like networks into ventricular models. Although many methodologies have been employed in the creation of these networks, for almost all modeling efforts so far, the Purkinje network has been manually or artificially generated rather than being based directly on anatomical data. In several studies, relatively simple Purkinje networks have been constructed to mimic known anatomical features of the Purkinje network, such as its position and coverage of the endocardial surfaces. Berenfeld and Jalife [9] created a Purkinje structural model by manually drawing a Purkinje network and projecting it onto the endocardial surface. The exact procedure by which this was accomplished was not detailed extensively, nor was it the focus of their work; rather, their goal was the creation of a structure having Purkinje-like characteristics, such as extent and area of the endocardial surface covered by the network. Clements and Vigmond [10, 11] developed a procedure by which a ventricular anatomical structure in the form of a triangulated mesh could be used to develop a Purkinje structural model. They began by isolating the portion of the model mesh constituting the endocardial surfaces. A sophisticated flattening algorithm then was used to map the faces in the endocardial meshes to the plane. Next, a manually drawn Purkinje network was laid onto the endocardial surfaces and the flattening transformation was inverted, yielding a three-dimensional Purkinje structure compatible with the initial cardiac structure.

Some studies have focused on modeling the Purkinje network structure to reproduce experimental observations regarding the normal activation sequence of the heart. Siregar et al. [12] constructed a combined model for both the ventricles and the Purkinje system in which the Purkinje network was generated primarily to activate regions of the ventricles observed experimentally to be sites of first activation. Simelius et al. [13] used a similar approach to design a model of the ventricular system that produced activations consistent with observed activation sequences and timings of the human heart, as well as with ECG data. Ten Tusscher and Panfilov [14] created a ventricular model incorporating a conduction system to investigate the role of the Purkinje network in normal and abnormal conduction. Their approach to generating a Purkinje network relied on user-specified terminal Purkinje fiber sites, which were then used to generate the full network. In all of these studies, the Purkinje network was generated to produce ventricular activation sequences consistent with experimental observations and with limited focus on anatomical realism.

The branching structure of the Purkinje network has been modeled using fractals in a number of studies. Abboud et al. [15] modeled the Purkinje network for use in reproducing ECG data through simulation as a self-similar branching tree structure, in which the initial His bundle connection of the network divided symmetrically into two smaller branches, which each further divided into smaller branches. Ijiri et al. [16] developed an algorithm based on the L-system for generating Purkinje networks given a cardiac structure and limited user input. Originally developed for modeling plant cells and structures, the L-system is a formal grammar that can be used in the implementation of a system for generating fractal-like structures based on simple rules. In this work, the L-system was used to generate a branching mesh structure that was confined to the endocardial surface starting from a number of user-defined terminal locations.

A key feature of current Purkinje structural models is that their construction was designed or guided by the modeler; all current methods have used either hand-drawn or computer-generated networks that were created to follow general principles characteristic of Purkinje networks. This shortcoming could be avoided by basing Purkinje structural models directly on anatomical data; unfortunately, it is not currently possible to capture the intact three-dimensional Purkinje network structure, and very little work has been done on using experimental data to directly create Purkinje structures. One recent study [17] has attempted to overcome this limitation by making use of imaging data of the Purkinje network in the form of photographs. Exposure to Lugol's solution preferentially darkens Purkinje fibers, thereby allowing the use of photographs to digitize the network. Such photographs were used to create two-dimensional Purkinje structural models that were incorporated into a model of the ventricles.

Our goal is to extend this recent approach to develop a method for accurate modeling of the three-dimensional Purkinje network structure. We will then incorporate these structures into anatomically realistic three-dimensional ventricle models, yielding a model for the ventricle-Purkinje conduction system. In contrast with existing work in the field, we seek to model the Purkinje structure through the direct use of imaging data taken from experimental photographs. Our modeling approach is to couple a ventricular model and a compatible Purkinje model. Since it currently is impossible to directly recover the full physical three-dimensional Purkinje structure, our first step is the development of a method for reconstructing this structure from photographs and anatomical data. We use this method to create the Purkinje structural models and then couple them with ventricular geometries to produce models for the entire ventricular conduction system.

An anatomically based model of the ventricle-Purkinje system should reproduce important features of the normal activation sequence of the ventricles. Specifically, the time and sequence of ventricular depolarization is fairly well known [18]. The goals we pursue in the development of our models are to achieve full activation of ventricles through a stimulus delivered by the Purkinje network and to chronologically activate the same regions within the ventricles as observed experimentally within the correct amount of time.

## 2. Methods

The first step in our development of ventricle-Purkinje models is the creation of anatomically realistic Purkinje structural models. We developed a technique to project the Purkinje network structures digitized from photographs onto the endocardial surfaces of three-dimensional anatomically realistic ventricle models. In doing so, the two-dimensional Purkinje structure is embedded in three-dimensional space superficial to the endocardium, yielding the three-dimensional Purkinje structural model. The original ventricular model along with the new Purkinje structural model then can be used as a domain in which cardiac model equations may be solved, yielding models for isolated excitable ventricular and Purkinje systems. The addition of two-way electrical coupling between the two models produces a combined model for the ventricle-Purkinje system.

### 2.1. Purkinje Structural Modeling

Our method begins with a technique we developed for projecting the two-dimensional Purkinje structure onto the endocardial surfaces of the ventricular data sets. Our strategy consists of two steps: first, map the texture image onto a surface that approximates the target surface; second, map from the approximating surface to the target surface. We use right cylinders as our approximating surfaces and extract the endocardial surfaces of the ventricle models as thin shells. The anatomical morphology of the endocardial surfaces and their orientation in the data sets used lend themselves quite well to this cylindrical approximation.

#### 2.1.1. Purkinje Network Photographs

**Figure 1:**
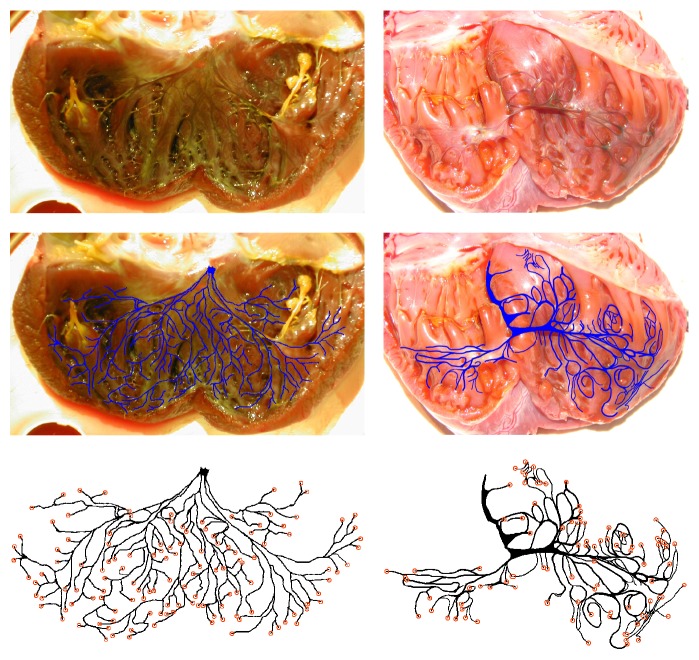
Top: photographs from experiments in which the left ventricle (left) and right ventricle (right) were dissected and treated with Lugol's solution, which stained the Purkinje network darker than surrounding tissue. The Purkinje network is visible to the eye. The left bundle branch is visible in the center at the top of the photograph, which corresponds to the septum wall. Middle: visible Purkinje fibers are manually traced in an image-editing program; the resulting network structure is overlaid for comparison onto the photograph from which it was extracted. Left: left ventricle. Right: right ventricle. Bottom: the resulting digitized Purkinje structures for the left (left) and right (right) ventricles. Coupling sites, located at the ends of the fibers, are indicated by orange circles.

The two-dimensional Purkinje structures were extracted from digital photographs of canine Purkinje networks after the ventricles had been dissected and treated with Lugol's solution, which preferentially stains Purkinje fibers darker than the surrounding cardiac muscle. In our work we use Purkinje networks recovered from left and right canine ventricles. The top panel of Figure 1 shows photographs of the dissected ventricles facing the endocardium. Following the collection of the photographs, the networks were digitized manually by tracing the darkened fibers in an image-editing program. Finally, coupling sites were added at the apparent end of each Purkinje fiber. The Purkinje network digitization used in our work for the left ventricle is the same as that used in [17]. The left network had a total of 130 coupling sites and the right network had 100 coupling sites. Due to the dissection method of the right ventricle, network connections were severed, and we did not attempt to match severed fibers across the dissection incision. Figure 1 (middle) shows the digitized Purkinje networks overlaid onto the experimental photographs from which they were extracted, and the bottom panel shows the isolated digitized Purkinje networks.

#### 2.1.2. Texture-Mapping Procedure

**Figure 2:**
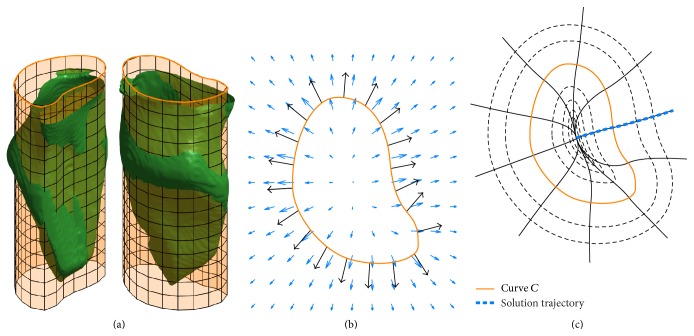
(a) The endocardial surface (green) shown with the curve *C* (orange) and associated cylinder that approximates the surface. (b) The curve *C* (orange) and a discrete sampling of the outward-pointing unit normal vector (black) to the curve shown alongside the induced vector field $\vec{V}$ (blue). (c) The induced curvilinear coordinate system. Plot shows lines of constant *s* (solid black), lines of constant *t* (dashed black), an individual solution trajectory for −*∞* < *t* < *∞* (dashed blue), and the original curve *C* (orange).

The procedure by which the two-dimensional Purkinje network structure is projected onto the endocardium begins with the selection of a curve *C* and associated cylinder to approximate the endocardial surface. Let *C* : [0, *L*] → *ℝ* ^2^ be a curve parameterized by arc length, where *L* is the total length of the curve. If *C* is a closed curve that is oriented in the counterclockwise direction, then taking the space *C* × *ℝ* we obtain a surface—a cylinder with base *C*. It is this cylinder that, in the context of the texture-mapping problem, is selected to approximate the surface to be textured.  Figure 2(a) shows an example cylinder chosen to approximate a given endocardial surface.

Define $\vec{N}(s)$, the outward-pointing unit normal vector to the curve *C* at $\vec{C}(s)$, by

(1) $$\begin{array}{c} \vec{N}\left(\;s\;\right)=\left[\begin{array}{cc} 0 & 1\\ -1 & 0 \end{array}\right]{\vec{C}}^{\prime }\left(\;s\;\right). \end{array}$$

Define the vector field $\vec{V}:{\mathbb{R}}^{2}\to {\mathbb{R}}^{2}$ by

(2) $$\begin{array}{c} \vec{V}\left(\;\vec{x}\;\right)=\frac{\int_{0}^{L}\vec{N}\left(s\right)d{\left(\vec{x},\vec{C}\left(s\right)\right)}^{-1}ds}{\int_{0}^{L}d{\left(\vec{x},\vec{C}\left(s\right)\right)}^{-1}ds}, \end{array}$$

where *d* is the distance function. $\vec{V}(\vec{x})$ forms a weighted average of $\vec{N}(s)$ at every point along $\vec{C}(s)$, where the weights are given by the inverse of the distance from $\vec{C}(s)$ to the point $\vec{x}$. As a consequence of this weighting scheme, we have that $\vec{V}(\tilde{p})\to \vec{N}(p)$ as $\tilde{p}\to p$ for all *p* ∈ *C*; that is, $\vec{V}$ converges to $\vec{N}$ near points on *C*. The reason for this convergence is that at a point $\vec{C}(s_{0})$ on the curve the two integrals in (2) diverge at *s*_0_.  Figure 2(b) shows an example approximating curve *C*, the outward-pointing unit normal vector $\vec{N}$, and a sampling of the resulting vector field $\vec{V}$.

Next, we introduce an artificial time parameter *t* for the purpose of obtaining solution trajectories of a particle moving through the vector field $\vec{V}$, according to the following system:

(3) $$\begin{array}{c} \frac{d\vec{x}}{dt}=\vec{V}\left(\;\vec{x}\;\right). \end{array}$$

In particular, for initial conditions $\vec{x}(0)=\vec{C}(s)$, we are interested in the solution $\vec{x}(t)$ for −*∞* < *t* < *∞*. Note that $\vec{x}(t)$ is normal to *C* at *C* because of the construction of *V*. We adopt the notation ${\left(s,t\right)}_{C}={\vec{x}}_{s}(t)$, where ${\vec{x}}_{s}(t)$ is the solution to the initial value problem given by (3) with initial condition ${\vec{x}}_{s}(0)=\vec{C}(s)$ for some *s* ∈ [0, *L*), for −*∞* < *t* < *∞*.

For curves *C* of interest, taking the family of solutions to (3) establishes a curvilinear coordinate system such that any point (*x*, *y*) ∈ *ℝ* can be given in curvilinear coordinates (*s*, *t*)_*C*_ for some *s* ∈ [0, *L*) and *t* ∈ *ℝ*. Note that ${\left(s,0\right)}_{C}=\vec{C}(s)$. The curvilinear coordinate system induced by an example curve *C* is shown in Figure 2(c).

We extend this curvilinear coordinate system in much the same way that cylindrical coordinates extend polar coordinates. Any point (*x*, *y*, *z*) ∈ *ℝ* ^3^ can be represented by (*x*, *y*, *z*) = (*s*, *t*, *z*)_*C*_ for some *s* ∈ [0, *L*), *t* ∈ *ℝ*. Now consider the surface

(4) $$\begin{array}{c} \left\{\;{\left(\;s,0,z\;\right)}_{C}:s\in \left[\;0,L\;\right),\;z\in R\;\right\}≅C\times R. \end{array}$$

This surface is the cylinder chosen to approximate the surface to be textured.

We now introduce the texture image that will be applied to the surface of interest. Let *T* : [0, *W*]×[0, *H*] → *𝒫* be the texture image with width *W* and height *H*. Here *𝒫* simply denotes the palette of which the texture image makes use. For a binary image we could have simply *𝒫* = {0,1}, or an 8-bit RGB image could be represented by a palette *𝒫* = [0,255]^3^ for the red, green, and blue components of the colors of the image.

We next position the texture image on this cylinder. We select some subset [*a*, *b*]×[*c*, *d*]⊆[0, *L*] × *ℝ*, a rectangular portion of the cylinder in which the texture image will reside. If the aspect ratio of the texture image should be preserved, we ensure that (*b* − *a*)/(*d* − *c*) = *W*/*H*. A point (*s*, 0, *z*)_*C*_ is assigned the texture value of the point (*u*_1_, *u*_2_), where

(5) u1s−ab−aW,u2=z−cd−cH.

That is, *T*(*u*_1_, *u*_2_) is the texture value of the point (*s*, 0, *z*)_*C*_.

This defines the texture value of a part of the cylinder. Using the curvilinear coordinate system we have established, we can extend this texture value definition along curves of constant *s* to define texture values for all points in *ℝ* ^3^. We now define an extension of our embedded two-dimensional texture image to three dimensions by constructing a function *F* : *ℝ* ^3^ → *𝒫*. Consider

(6) Fx,y,zTs,t,zC=Ts,0,zC=Fs−ab−aW,z−cd−cH.

Here *F* represents the two-dimensional Purkinje network structure projected onto the endocardial surface and extended perpendicular to the surface into three dimensions. Evaluating *F* within the domain of the endocardial surface yields the portion that lies within the thin-shell endocardial layer and thus the three-dimensional Purkinje network structure.

#### 2.1.3. Implementation Considerations for Purkinje Structural Modeling

Careful selection of the approximating curve *C* is important to achieve good results. In our work, we used closed Bezier splines as our approximating curves. Bezier curves are defined by a series of control points that control which points the curve passes through and the general shape of the curve; a Bezier spline is a piecewise-defined spline composed of multiple Bezier curves, which allows for a great deal of fine-tuning of the curve.

Evaluating $\vec{V}(p)$ involves evaluating three line integrals along *C* and so is computationally expensive. In order to reduce the computational cost and runtime of the algorithm, we first evaluated $\vec{V}$ on a numerical grid of evenly spaced points. To evaluate $\vec{V}$ at an arbitrary point, we interpolated this known grid of $\vec{V}$ values using the MATLAB function interp2.

Although any point (*x*, *y*) ∈ *ℝ* should have a corresponding coordinate (*s*, *t*)_*C*_ in the curvilinear coordinate system induced by *C*, closed-form expressions for converting between the two coordinate systems would be difficult or impossible to find. In practice what we wish to do is to find the (*s*, *t*)_*C*_ coordinates of many points (*x*, *y*). We solved system (3) with initial condition $\vec{x}(0)=\vec{C}(s)$ for many values of *s* to produce many solutions starting from evenly spaced points along *C*. We solved the system both forwards and backwards in time to sufficiently cover the domain of interest and then concatenated the solutions, resulting in solutions of (3) over −*∞* < *t* < *∞*. We then interpolated these solutions over the domain of interest to assign to every point (*x*, *y*) the value of *s* such that (*x*, *y*) = (*s*, *t*)_*C*_ for some *t*.

### 2.2. Coupled Ventricle-Purkinje Models

We next form models for the combined ventricle-Purkinje system by coupling a model of the ventricles and a Purkinje structural model.

#### 2.2.1. Ventricular Geometries

**Figure 3:**
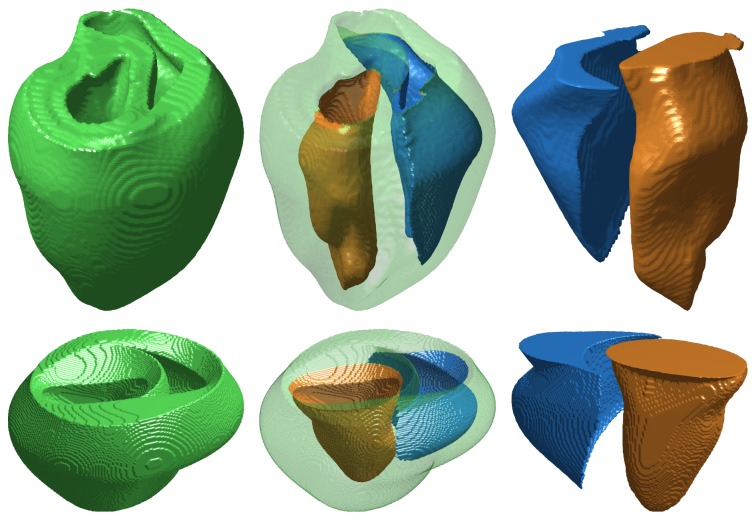
Isosurface plots of the rabbit (top) and canine (bottom) ventricular structures we used in our work. Left: posterior views of the ventricular structures. Center: transparent overlays of the epicardial surfaces, shown in green, aligned with the corresponding endocardial surfaces, shown for comparison and orientation. The left endocardial surfaces are shown in orange and the right endocardial surfaces are shown in blue. Right: alternate viewing angle of the isolated endocardial surfaces of the left and right ventricles.

The goal of this study was the development of a method for reconstructing the three-dimensional Purkinje network structure from two-dimensional photographs and full ventricular models. Although we had access to only canine Purkinje network photographs, we incorporated these network branches into both rabbit [19] and canine [20] ventricle models to demonstrate and test the generality of our methods. The rabbit ventricles are embedded in a rectangular numerical grid of size 138 × 130 × 152 with a uniform grid spacing of 0.025 cm. Vetter and McCulloch [19] also obtained fiber orientation data, which we used in our model. The ventricles are embedded in a rectangular numerical grid of size 400 × 320 × 320 with a uniform grid spacing of 0.025 cm.  Figure 3 shows isosurface plots of the rabbit and canine ventricle models, with the epicardial and endocardial surfaces highlighted in several views.

#### 2.2.2. Model Development

**Figure 4:**
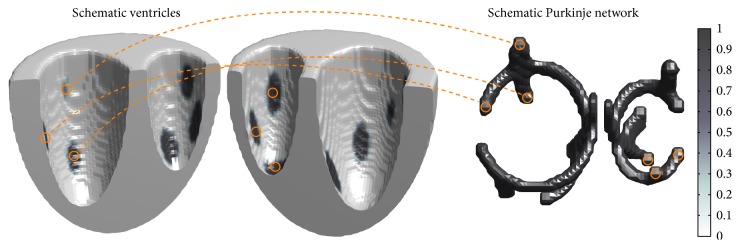
Schematic visualization of a 3D-3D model. The structure is a simplified contrived structure used for illustrative purposes only and is not anatomically realistic; the conduction system shown is highly simplified and the number of coupling sites depicted here is much lower than in real networks to facilitate visualization and testing. Orange dashed lines show several of the electrical coupling sites between the Purkinje network branches and the ventricle.

We developed two models for the coupled ventricle-Purkinje system. The first, which we have termed the 3D-3D model, represents both the ventricular and Purkinje systems in three dimensions. The 3D-3D model is the natural extension of the ventricle-Purkinje model developed in [17].  Figure 4 shows a schematic visualization of how coupling is implemented in the 3D-3D model; it illustrates the constituent models and where electrical coupling between the models occurs. Whereas the model of [17] coupled to a two-dimensional ventricular and a two-dimensional Purkinje model, the model presented here couples a three-dimensional ventricular and a three-dimensional Purkinje model. The key similarity between these two approaches is that both constituent models are computed on the same numerical grid. This leads to a straightforward means of achieving electrical coupling: defining a number of grid points as being two-way coupled, thereby allowing the two models to influence each other at a number of discrete sites.

**Figure 5:**
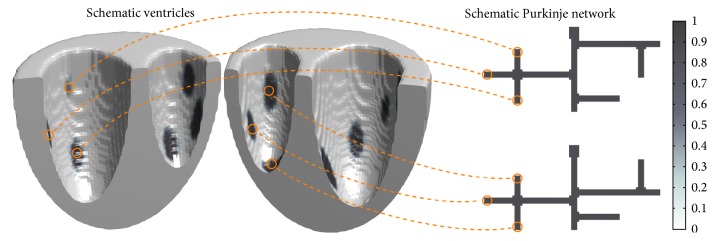
Schematic visualization of a 3D-2D model. The structure is a simplified contrived structure used for illustrative and testing purposes only and is not anatomically realistic. To aid in visualization, the number of electrical coupling sites shown is much lower than in real networks. Orange dashed lines show several of the electrical coupling sites between the Purkinje network branches and the ventricle.

The second model, which we refer to as the 3D-2D model, represents the ventricles in three dimensions and the Purkinje network in two dimensions.  Figure 5 shows a schematic of the components of the 3D-2D model and shows how the models are electrically coupled. This modeling approach differs in an important way from that of [17] and the previously discussed 3D-3D model in that the ventricular and Purkinje models do not occupy the same coordinate space. The implementation for both constituent models remains largely the same as that of the 3D-3D model, but the means by which the two models are coupled together is significantly different. Whereas, in the previous approach, both the ventricles and the Purkinje network were represented in the same space and solved on the same numerical grid, here they are not. Thus, the straightforward means of defining some grid points as being two-way coupled cannot be applied to couple the models together. In our implementation, coupling is implemented between a list of locations of each coupling site in the 2D Purkinje network and the corresponding locations on the ventricular surface of that point under the texture map. Programmatically, model variables at grid points in the list are updated in a second phase after updating all grid points under whichever numerical method is chosen to implement the cardiac reaction-diffusion equations.

The development of the 3D-2D model was motivated by the fact that the Purkinje network is largely confined to the endocardial surface and therefore is effectively a two-dimensional structure embedded in three dimensions. We hypothesized that a model that represented the Purkinje network as being two-dimensional would reproduce all important dynamics observed in an analogous three-dimensional Purkinje network and that the former model could provide several benefits. One such benefit is computational cost: representing the sparse Purkinje network structure in two rather than three spatial dimensions greatly reduces the number of node points in the numerical grid and thus reduces the runtime of the model. Second, numerical solution of the Purkinje model on a numerical grid that is independent of the grid used for the ventricular model allows for the Purkinje network to be represented at a different resolution than that of the ventricular model. The fine detail of the Purkinje structure thus can be represented more accurately without having to refine the resolution of the ventricular model.

#### 2.2.3. Implementation Considerations for Coupled Models

In data sets containing the full ventricular structure, it is necessary to isolate a thin shell (the endocardial layer) before proceeding with texture-mapping. We use a phase field representation of the ventricles [21] as part of this process. To isolate a thin shell, we begin by isolating the ventricular chamber, based on taking that volume of the phase field below a given threshold (such as *ϕ* = .5) within the chamber. This results in a Boolean matrix of grid points that lie within the chamber. We then apply a three-dimensional convolution with a spherical kernel and take all entries with a value greater than 1, resulting in a matrix of the same size. This resulting matrix represents a volume expanded from that of the ventricular chamber; it overlaps the ventricular tissue by a width of approximately the radius of the spherical kernel. A thin shell is obtained by taking the nonzero entries in this matrix that also satisfy some threshold value in the phase field.

### 2.3. Cardiac Cell Electrophysiology Model

The models we have developed are structural rather than electrophysiological. In order to study the effect of the Purkinje structure on ventricular activation and arrhythmia, a cardiac electrophysiological model must be selected to represent the excitability of the cardiac tissue. In our work we model the excitability of tissue by the monodomain equation

(7) $$\begin{array}{c} \frac{\partial V}{\partial t}=-I_{\text{ion}}+\nabla \cdot \left(\;D\left(\;x\;\right)\nabla V\;\right). \end{array}$$

Here *V* represents the cell membrane potential or voltage, **D** is a tensor defining the local orientation of the muscle fibers as well as the diffusivity along and against the fiber direction, and *I*_ion_ is the sum of the various transmembrane ion currents, dictated by a single-cell cardiac electrophysiology model.

Many cardiac electrophysiology models of greatly varying complexities have been developed [22]. The selection of the appropriate model depends on a number of factors, such as the aim of the study and computing resources available. The model choice can affect many dynamics relating to electrical wave behavior and arrhythmias, such as conduction velocity, alternans, and spiral- and scroll-wave dynamics. Our goal is the validation of our work as a suitable model for the ventricular conduction system, and so we sought to synchronize activation times of the ventricular tissue with those found experimentally. To represent activation times realistically, only a simple cardiac model is needed to capture the key aspects of the depolarization wavefront. For this reason, we used the two-variable Mitchell-Shaeffer model [23] to represent both cardiac muscle cell and Purkinje fiber cell dynamics. To represent the significantly faster propagation of excitation in Purkinje cells as compared to cardiac muscle cells, the diffusivity in the Purkinje model was made 20 times greater than in the ventricular model.

### 2.4. Numerical Parameters

In the 3D-3D model, all constituent models are computed on the same numerical grid, with the same uniform grid spacing used in each model. In the 3D-2D model, models are computed on potentially different and sometimes nonuniform grids, and so consideration must be given to ensure that grid spacing is chosen to accurately represent the physical situation.

In cases where the aspect ratio of the digitized Purkinje network was not preserved, we implemented the 2D model with nonuniform grid spacing. Where a subset of the approximating cylinder given in the curvilinear coordinate system by [*a*, *b*]×[*c*, *d*] was selected as the portion of the cylinder on which the texture image was to reside, we defined the grid spacing Δ_*P*_ *x* and Δ_*P*_ *y* of the Purkinje network by

(8) ΔPxb−aWΔVx,ΔPy=d−cHΔVx,

where Δ_*V*_ *x* is the grid spacing used in the ventricular model (which we always took to be uniform) and *W* and *H* are the width and height of the digitized Purkinje network image, respectively.

## 3. Results and Discussion

### 3.1. Texture-Mapping

By applying our texture-mapping techniques using the digitized Purkinje structures and ventricular models, we were able to produce three-dimensional Purkinje structural models.

**Figure 6:**
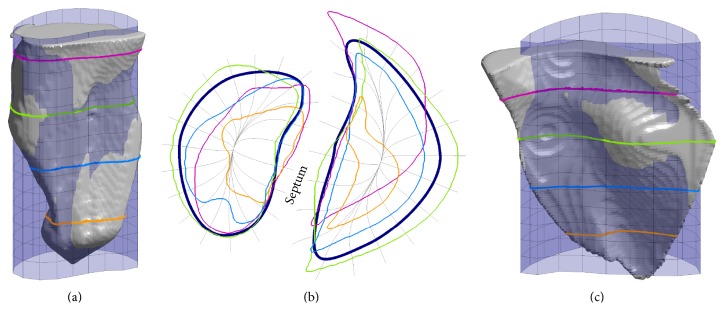
The left and right rabbit ventricular endocardial surfaces and associated curve and cylinder chosen to approximate the surface. (a, c) The left and right, respectively, endocardial surfaces (grey), the associated approximating cylinder (transparent), and surface contour lines taken at various levels of the surface. Both views of the surfaces are facing the septum wall. (b) The approximating curves and the resulting curvilinear coordinate system plotted against the same contour lines taken from the endocardial surfaces.

**Figure 7:**
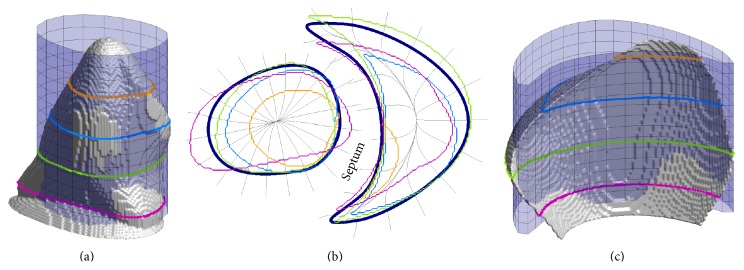
The left and right canine ventricular endocardial surfaces and associated curve and cylinder chosen to approximate the surface. (a, c) The left and right, respectively, endocardial surfaces (grey), the associated approximating cylinder (transparent), and surface contour lines taken at various levels of the surface. Both views of the surfaces are facing the septum wall. The views of the surfaces are inverted vertically for better viewing. (b) The approximating curves and the resulting curvilinear coordinate system plotted against the same contour lines taken from the endocardial surfaces.

The curves and associated cylinders chosen to approximate the ventricular endocardial surfaces are shown in Figures 6 and 7 for the rabbit and canine ventricles, respectively. From the plots, it can be seen at various cross sections perpendicular to the cylinder axis that the surfaces are well approximated. Particularly near the septum wall, the cylinders closely approximate the true endocardial surface. Near the apex, the cylindrical approximations do not match as well, especially for the right ventricles. In practice, we found this not to be a limitation, because the Purkinje network structure did not extend into these regions.

We developed the texture-mapping technique with the goal of creating anatomically realistic Purkinje network structures; as part of our validation of the network structures we obtained, we compared them to the experimental photographs from which the Purkinje network structures were extracted. The position and extent of coverage on the endocardial surfaces are important features of the network, particularly in the context of our goal of anatomical realism, but also for achieving realistic activation sequences of the ventricles. Figure 1 shows the original experimental photographs and digitized Purkinje networks. The positioning of each branch of the Purkinje network is most easily described in reference to the His bundle connection, or bundle branch. Physiologically, the bundle branch is the source of all Purkinje fibers and begins within the septum wall.

In the left ventricle photographs, the bundle branch connection is located near the center of the endocardium. The means by which the left ventricle was dissected involved a cut in the free wall opposite the septum, which is why the bundle branch is in the center of the resulting photograph. The Purkinje network spreads outward from the bundle branch, and fibers evenly cover most of the endocardial surface. The network is quite symmetric in its coverage, with fibers reaching the free wall (along which the dissection was made) from both directions. When texture-mapping the left ventricle endocardial surfaces, we aimed to reproduce these physiological features. The networks were positioned to cover the entire endocardium, and the bundle branch was positioned at the center of the septum wall.

**Figure 8:**
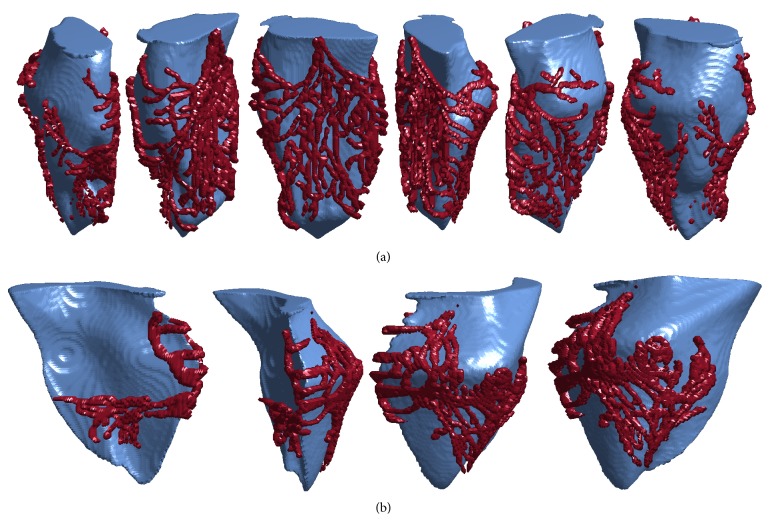
Visualizations of the three-dimensional Purkinje structures (red) and corresponding endocardial surfaces (blue) for the rabbit ventricles. (a) The left endocardial surface and Purkinje network branch. The Purkinje network covers almost all of the endocardial surface, which coincides with observations from dissections. (b) The right endocardial surface and Purkinje network branch. The Purkinje network is oriented on the endocardial surface such that the His bundle connection is aligned with the septum-free wall seam, as observed in the dissected network.

**Figure 9:**
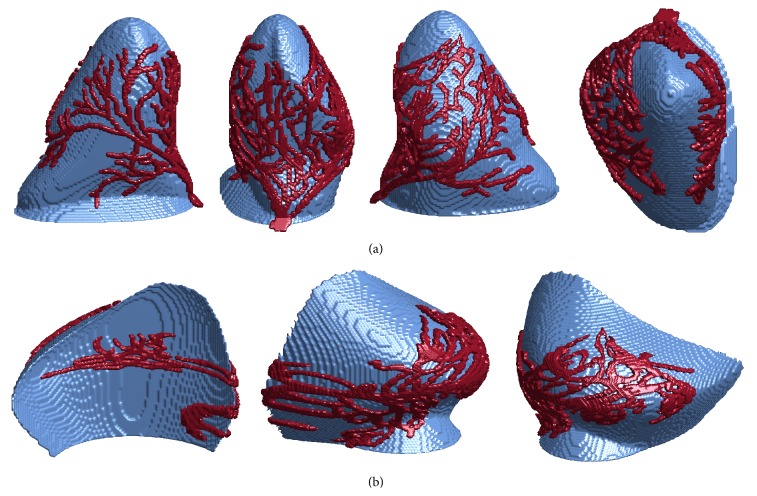
Visualizations of the three-dimensional Purkinje structures (red) and corresponding endocardial surfaces (blue) for the canine ventricles. Here the view is vertically inverted for more convenient viewing. (a) The left endocardial surface and Purkinje network branch. The Purkinje network covers almost all of the endocardial surface, which coincides with observations from dissections. (b) The right endocardial surface and Purkinje network branch. The Purkinje network is oriented on the endocardial surface such that the His bundle connection is aligned with the septum-free wall seam, as observed in the dissected network.

Figure 8 shows the Purkinje structure models generated with texture-mapping of the left Purkinje branch and left ventricle for the rabbit ventricles. The aspect ratio of the digitized network was altered significantly so that the extent of the texture-mapped network would cover nearly all of the endocardial surface, so as to coincide with experimental observations of Purkinje networks, as in Figure 1. The network was positioned so that the bundle branch originated near the center of the septum wall, and the network wrapped around the entire endocardial surface.  Figure 9 shows the texture-mapping-generated Purkinje structure models for the canine left Purkinje branch and left ventricle; here the aspect ratio of the digitized network was able to be preserved while still achieving coverage of nearly all of the endocardial surface. As in the rabbit left ventricle, the network was positioned so that the bundle branch connection coincided with the center of the septum wall.

The dissection method used for the right ventricle differed significantly from that used for the left ventricle: the right ventricle was cut along a “seam” between the septum and free wall. Figure 1 shows the endocardial surface corresponding to the septum on the left and the free-wall endocardial surface on the right. The overall network structure of the right branch is much less symmetric than that of the left branch, both visually and from a physiological perspective. The right network branch still begins at the bundle branch, but in this case the bundle branch is not positioned at the middle of the septum wall. Instead, it is positioned at the other seam between the septum and free wall. The network coverage of the septum endocardial surface is limited, with fibers only appearing near the apex. The free wall is covered extensively in network fibers much like the left ventricle.

Figures 8 and 9 show the Purkinje structure models generated with texture-mapping for the right ventricle-Purkinje branch of the rabbit and canine ventricles, respectively. In both networks, the Purkinje network was aligned and the aspect ratio altered so that the extent and placement of the Purkinje network coincided with experimental observation: the bundle branch connection was aligned with the septum-free wall seam; the portion of the network on the septum wall extended across the entire lower septum wall and did not encroach on the other side of the free wall; and the portion of the network on the free wall wrapped around the entire free wall without encroaching on the septum wall.

Thus, based on visual inspection alone, the texture-mapped Purkinje networks provide a reasonable representation of the Purkinje network with respect to apparent anatomical realism. Key features of the Purkinje network as observed from experiments are closely reproduced in our models.

### 3.2. Validation of Coupled Ventricle-Purkinje Models

**Figure 10:**
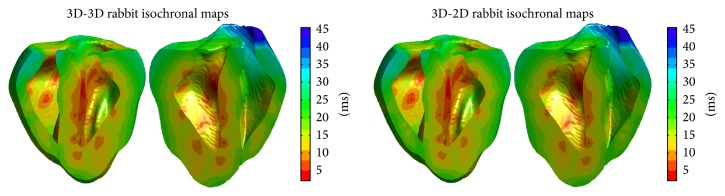
Isochronal plots showing activation times of the rabbit ventricles in the 3D-3D and 3D-2D models. Colors indicate the time at which each point in the tissue experienced depolarization, with zero corresponding to the first activation within the entire ventricles.

As our next validation step, we aimed to compare simulated activation times in a combined ventricle-Purkinje model with activation times observed in experiments. Toward this end, the three-dimensional Purkinje structures recovered through texture-mapping were used as the conduction system in the development of models of the combined ventricle-Purkinje system. Our goal was to create a model that reproduced key features of the real cardiac conduction system. In particular, we aimed to ensure that our model reproduced the overall progression of the total depolarization (activation) of the ventricles, termed the activation sequence, which has been well characterized [18]. Key features of the activation sequence are those areas that are the first to depolarize: activation begins in the left ventricular endocardial surface, particularly in several isolated spots, in the middle of the septum wall and in the upper portion of the free wall. The last region to depolarize is the right ventricular free wall.  Figure 10 shows isochronal plots of the activation times achieved in our models. Importantly, the left ventricular endocardium contains some of the sites of earliest activation, particularly on the septum. The right ventricular free wall is also the last region to depolarize, matching with experimental observations. In addition to achieving a realistic activation sequence, the entire activation of the ventricles occurs in realistic time [18, 24].

We conclude that the ventricular activation sequences produced by the 3D-3D and 3D-2D models match well with experimental observations. The overall activation time, as well as sequences of primary activation sites produced by our models, coincides well with experimental observations.

**Figure 11:**
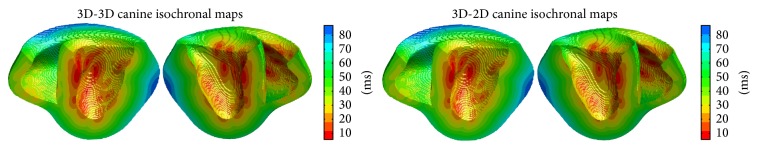
Isochronal plots showing activation times of the canine ventricles in the 3D-3D and 3D-2D models. Colors indicate the time at which each point in the tissue experienced depolarization, with zero corresponding to the first activation within the entire ventricles.

Figure 10 shows isochrone plots of the activation of the rabbit ventricles in the 3D-3D and 3D-2D models, and Figure 11 shows results for the canine ventricles. In all of the models, the first sites to depolarize were located on the endocardium, corresponding to ventricle-Purkinje coupling sites. Further depolarizations induced by the Purkinje network occurred as activation spread from these sites throughout the entire ventricles.

It is apparent from the plots that activation in the 3D-3D and 3D-2D models for the same ventricles is qualitatively very similar, with some minor differences. Quantitatively we found in the rabbit models that in relation to the start of the stimulus protocol the 3D-3D model was faster to activate than the 3D-2D one by about 4 ms overall, mainly due to the Purkinje network activating more quickly in the 3D-3D model. The 3D-3D model exhibited a first ventricular depolarization after 9 ms, whereas the 3D-2D model took 13 ms to achieve its first depolarization. In the canine models, first ventricular activations occurred at the same time, at 11 ms after the start of the stimulus protocol.

Visualization of differences in ventricular activation between the two models is desirable to understand implications of the distinct model constructions. Because the ventricular structures are identical in both models, we can visualize differences in activation by plotting the difference in activation times at each point in the ventricles.

Differences in activation sequences and timings between the two models can be measured in several ways. Since the two models were subjected to the same stimulus procedure, the difference in activation times between the two models at every point in the ventricles can be directly compared to measure the activation time differences from the beginning of the stimulus procedure applied to both models. Taking $A_{3}(\vec{x})$ and $A_{2}(\vec{x})$ to be the activation times in the 3D-3D and 3D-2D models, respectively, of a given point $\vec{x}$ in the ventricles measured from the start of the stimulus protocol, this measure is given by $A_{3}(\vec{x})-A_{2}(\vec{x})$. It is also possible to measure differences in activation based on the first activation within the ventricles. Calling *f*_3_ and *f*_2_ the time from the beginning of the stimulus protocols to the first activations in the 3D-3D and 3D-2D models, respectively, this difference is given by $\left(A_{3}(\vec{x})-f_{3})-(A_{2}(\vec{x})-f_{2}\right)$. Finally, the activation of the ventricles in both models can be compared by attempting to minimize differences in activation times at each point in the tissue according to a criterion such as least squares—that is, by attempting to minimize

(9) $$\begin{array}{c} \underset{\vec{x}\in V}{\sum }{\left(\;A_{3}\left(\;\vec{x}\;\right)-A_{2}\left(\;\vec{x}\;\right)+c\;\right)}^{2} \end{array}$$

by choice of *c*, where *V* is the entire ventricular domain and *A*_3_ and *A*_2_ are evaluated over *V*. Here *c* is an offset parameter tuned to minimize discrepancy in activation times between the two models.  Table 1 summarizes these comparative schemes, with differences taking 3D-3D activation times minus 3D-2D activation times. Note that because first activations in the two canine models occurred at the same time relative to the start of the stimulus protocol, the measures given according to stimulus start and first ventricular activation are the same.

**Figure 12:**
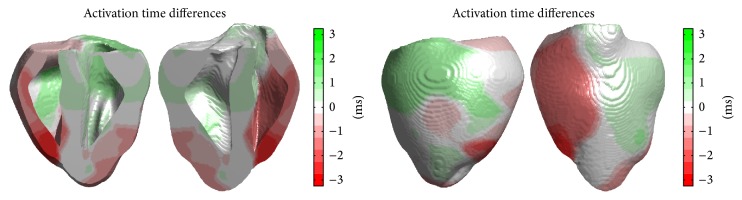
Comparison of activation times in the rabbit ventricles in the 3D-3D and 3D-2D model implementations. Plots show the difference in activation times of the ventricles between the 3D-3D and 3D-2D models, with a positive difference indicating the 3D-2D model activated earlier than the 3D-3D model.

**Figure 13:**
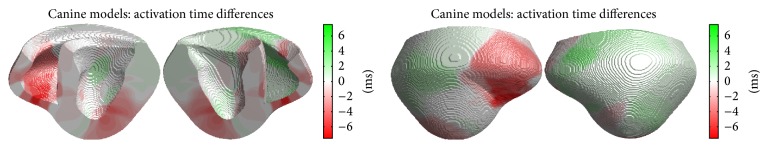
Comparison of activation times in the canine ventricles in the 3D-3D and 3D-2D model implementations. Plots show the difference in activation times of the ventricles between the 3D-3D and 3D-2D models, with a positive difference indicating the 3D-2D model activated earlier than the 3D-3D model.

Figures 12 and 13 show differences in activation times between the 3D-3D and 3D-2D models for the rabbit and canine systems, respectively; in the plots, activation times are offset to minimize the root mean square difference. The well-defined regions of earlier and later activation of the models indicate that the two- and three-dimensional Purkinje network structures in the two models have a degree of nonuniform distortion. There are several reasons why such differences may be expected. The three-dimensional Purkinje structure in the 3D-3D model will be a distorted version of the original two-dimensional Purkinje structure, due to deviations in the endocardial surface from the cylindrical approximating surface. Distances perpendicular to the axis of the cylinder will shorten in regions of the surface that lie within the cylinder and lengthen in regions outside of the cylinder. Distances parallel to the axis of the cylinder will lengthen only. Despite the differences in construction, the two models show very similar activation sequences.

In our program implementations, the 3D-2D models ran significantly faster than the corresponding 3D-3D models. Specifically, the 3D-2D model was about 2.5 times faster than the 3D-3D model for the canine geometry and about 4.5 times faster for the rabbit geometry. The overall faster speed of the 3D-2D model implementations is most likely due to the reduced number of computational nodes in the two-dimensional Purkinje representation; grid points excluded from the computational domain because of a sufficiently low phase field value may be skipped, providing significant speedup. The difference in observed speedup factors between the canine and rabbit geometries arises from differences in the canine and rabbit ventricular structure sizes and morphologies.

### 3.3. Limitations

There are several important limitations regarding our method and results. First, we assume that the Purkinje network is superficial, which is likely to be a reasonable assumption in species like canines and humans but not for other species, such as ungulates, in which the Purkinje network is known to penetrate more deeply into the myocardial wall [6]. Our method makes use of this assumption in two ways. First, the means by which we retrieve the two-dimensional network structure relies on the anatomical fact that the Purkinje network is superficial. Second, the means of reconstructing the three-dimensional network structure through texture-mapping necessarily projects the network structure onto the endocardial surface. Little is known about the detailed intramural structure of Purkinje fibers in species with more fully three-dimensional network structures. However, our technique could be extended in a straightforward manner to account for the presence of Purkinje fibers in the wall by extending fibers from their coupling sites in a direction normal to the endocardial surface, for example.

The full extent of the Purkinje network is not well known; our work dealt with the network structure that was readily captured in high-resolution photographs obtained through experimental dissection, but small Purkinje fibers may not be captured in such photographs or may be too fine to adequately represent using finite-difference methods. In our work with macroscopic Purkinje structures we faced issues with representing structures with fine detail on comparatively coarse grids, and so representing even finer-detail networks may pose a problem to advancing these methods. This limitation is somewhat lessened in the 3D-2D model, where the ventricular and Purkinje structural models may be represented at different resolutions.

Several limitations stem from the fact that our texture-mapping procedure relies heavily on the approximation of the endocardium by a cylinder. In our work with a rabbit ventricular structure, we found this approximation to be reasonable and were able to apply our texture-mapping procedure successfully. The approximation was more robust in the left ventricle than the right ventricle, where it was acceptable overall, but regions of the surface that were poorly approximated by the chosen cylinder were present. We were able to apply the texture-mapping procedure to obtain networks that agreed well with experimental observations; for other data sets, this approximation may be unsuitable. It is also the case that the cylindrical approximation of the ventricles requires the alignment of the cylinder axis with one of the main coordinate axes of the computational box in which the anatomical structure is embedded. Some data sets may not be oriented appropriately to readily allow the approximation of the ventricles by cylinders. One possible means of overcoming this problem is the resampling of the anatomical structure through a rotational transformation.

While aligning the flat Purkinje network images with the approximating cylinders, we did not seek to preserve aspect ratios; rather, we selected the area for the image to lie within to match key physical characteristics observed from dissection. There are several reasons why changing the aspect ratio as we did might result in a more realistic three-dimensional Purkinje network structure. First, the Purkinje network images and ventricular structures we used were obtained in separate studies from different hearts and, in some cases, different species. Structural differences in size and shape between the endocardial surfaces will naturally lead to distortion. Second, the dissection method used on the ventricles did not allow for the endocardial surfaces to be laid perfectly flat. This means that the photographs, which we assumed to capture the flattened endocardial surface, actually represented stretched, compressed, and curved tissue, which led to distortions in distances and areas in the Purkinje network structure.

To assess the robustness of the modeling technique, we performed a preliminary sensitivity analysis on the rabbit models by generating perturbed models and comparing activation sequences. The perturbed models utilized the same ventricular geometries but slightly adjusted Purkinje structural models; these models were created by applying the texture-mapping procedure with the texture images at a slight offset from the original models. For our analysis, we offset each network image by approximately 1 mm rotationally around the approximating cylinder in each direction to produce four perturbed networks. We found that total activation time within the ventricles was within about 5 ms, with the most noticeable differences occurring near the coupling sites and on the endocardium.

### 3.4. Future Work

Our work can be extended in several ways to provide greater realism, robustness, and efficiency.

To obtain two-dimensional Purkinje structures from experimental photographs, we manually traced visible Purkinje fibers in an image-editing program. Algorithms for isolating features in images from the field of computer vision may be adapted to automate this task and could possibly capture more of the full network structure.

The method by which the ventricles were dissected can potentially sever fiber connections, altering the perceived structure of the Purkinje network when photographed. In our work we did not attempt to make use of this fact, but in principle fiber connections could be matched across the incision, potentially leading to better digitization of the Purkinje network. A further step could be the use of nonlinear image stitching to associate fiber connections from one side of the incision with the other and to potentially reconstruct more of the connected Purkinje network.

In the texture-mapping procedure, we begin by approximating the target surface with an approximating cylinder. In principle, our method should be extensible to arbitrary approximating surfaces. This would require a significant change in methods; specifically, the projection of the flat two-dimensional Purkinje network structure onto the approximating surface would no longer be a straightforward *C* ^1^ isometric embedding.

In our study we had access to a limited number of Purkinje network images: one dataset each for canine left and right network branches. We used these canine Purkinje network structures to construct Purkinje structural models for both rabbit and canine ventricles and subsequently achieved realistic activation times in our ventricle-Purkinje models. To create more realistic structural models, Purkinje network images and ventricular models from the same species should be used.

In the future, it will be important to assess differences in Purkinje structures across different hearts and different species along with how these differences affect normal ventricular activation and ventricular arrhythmias. The method we developed can be applied to produce Purkinje network structures for any species with superficial Purkinje networks, which will facilitate this work.

## 4. Conclusions

We presented a novel method for reconstructing the Purkinje network structure in three dimensions. The method directly uses imaging data taken from experiments and is the first such effort to model the three-dimensional Purkinje network structure. Previous studies that involved the construction of a Purkinje network either did so as a component of other work and did not focus on the construction procedure, were not based directly on experimental data, or generated artificial networks.

Our method makes the recovery of Purkinje network structures that are compatible with real anatomical ventricular structures possible, enabling the creation of models for the combined system. We have presented the first ventricular-Purkinje structural models where both the ventricles and Purkinje network were based directly on data taken from experiments. We implemented two of these models and were able to reproduce simulated ventricular activation sequences that closely match experimental observations.

Our model implementations depolarize the ventricles solely through the Purkinje network and achieve realistic activation times and an activation sequence that agrees well with experimental observations. This suggests that our models and modeling approach accurately capture essential characteristics of the ventricular conduction system.

The development of these models is an important step forward in the investigation of cardiac arrhythmias. These model formulations can facilitate future studies of the contributions of the Purkinje network to the maintenance and initiation of ventricular arrhythmias, as well as investigations of arrhythmias related to the cardiac conduction system such as bundle branch block.

## Figures and Tables

**Table 1:** Summary of differences in activation times between the 3D-3D and 3D-2D rabbit and canine models according to three different schemes: measured with respect to the start of the stimulation protocols, first ventricular activation, or to minimize the root mean square difference. Measurements are given in milliseconds. For signed quantities, the 3D-2D activations times were subtracted from the 3D-3D activations times.

Rabbit models	Stimulus start	First ventricular activation	Minimizing RMS
Root mean square difference	4.4	0.9	0.7
Standard deviation of differences	0.7	0.7	0.7
Minimum difference	−7.2	−3.3	−2.7
Maximum difference	−2.0	+1.9	+2.4

Canine models	Stimulus start	First ventricular activation	Minimizing RMS

Root mean square difference	1.4	1.4	1.4
Standard deviation of differences	1.4	1.4	1.4
Minimum difference	−6.8	−6.8	−6.5
Maximum difference	+2.8	+2.8	+3.1
